# Consumption Frequency of Foods Away from Home Linked with Higher Body Mass Index and Lower Fruit and Vegetable Intake among Adults: A Cross-Sectional Study

**DOI:** 10.1155/2016/3074241

**Published:** 2016-01-26

**Authors:** Rebecca A. Seguin, Anju Aggarwal, Francoise Vermeylen, Adam Drewnowski

**Affiliations:** ^1^Division of Nutritional Sciences, Cornell University, 412 Savage Hall, Ithaca, NY 14853, USA; ^2^Center for Public Health Nutrition, University of Washington, 330 Raitt Hall, Seattle, WA 98195, USA; ^3^Division of Nutritional Sciences, Cornell University, B07 Savage Hall, Ithaca, NY 14853, USA; ^4^Nutritional Sciences, University of Washington, 305 Raitt Hall, Seattle, WA 98195, USA

## Abstract

*Introduction*. Consumption of foods prepared away from home (FAFH) has grown steadily since the 1970s. We examined the relationship between FAFH and body mass index (BMI) and fruit and vegetable (FV) consumption.* Methods*. Frequency of FAFH, daily FV intake, height and weight, and sociodemographic data were collected using a telephone survey in 2008-2009. Participants included a representative sample of 2,001 adult men and women (mean age 54 ± 15 years) residing in King County, WA, with an analytical sample of 1,570. Frequency of FAFH was categorized as 0-1, 2–4, or 5+ times per week. BMI was calculated from self-reported height and weight. We examined the relationship between FAFH with FV consumption and BMI using multivariate models.* Results*. Higher frequency of FAFH was associated with higher BMI, after adjusting for age, income, education, race, smoking, marital status, and physical activity (women: *p* = 0.001; men: *p* = 0.003). There was a negative association between frequency of FAFH and FV consumption. FAFH frequency was significantly (*p* < 0.001) higher among males than females (43.1% versus 54.0% eating out 0-1 meal per week, resp.). Females reported eating significantly (*p* < 0.001) more FV than males.* Conclusion*. Among adults, higher frequency of FAFH was related to higher BMI and less FV consumption.

## 1. Introduction

There has been a notable shift in eating culture in the past 40 years. Since the late 1970s, consumption of foods prepared outside the home has steadily grown, from one-sixth to almost one-third of an individual's daily dietary intake [1–4]. A 2012 analysis of 2007-2008 National Health and Nutrition Examination Survey (NHANES) data found that 41% of adults consumed foods and/or beverages from fast food-type restaurants and 27% from full-service restaurants during the previous 24 hours [2]. With fewer meals being prepared at home, the overall quantity of calories has increased while nutritional quality has declined.

Not surprisingly, individuals who consume more FAFH are reported to have poorer diet quality. Higher fruit and vegetable intake is associated with better dietary quality [5, 6], and some studies have found an inverse relationship between frequency of fast food use and daily servings or meal density of fruit and vegetables [7–12]. The Healthy Eating Index (HEI) is a measure of diet quality, with total fruit and total vegetable intake as two of the main components of this composite measure [13]. Economic Research Service Reports using the 1994–96 Continuing Survey of Food Intakes by Individuals (CSFII) and 2003-04 NHANES data found that one meal away from home lowered the daily HEI score enough to shift the average adult's diet quality from a classification of fair to poor [12, 14]. Another study using the CSFII 1994–96 data reported that more days of fast food consumption resulted in increased energy and macronutrient intakes and decreased micronutrient density [15]. More specifically, eating fast food was associated with lower intakes of vitamin A, carotene, vitamin C, calcium, and magnesium, high amounts of nondiet carbonated soft drinks, and insignificant amounts of fruit [15]. Other studies have found that those who consume FAFH more frequently have higher intakes of total energy, total fat, saturated fat, cholesterol, sodium, sugar-sweetened beverages, and sugar and lower intakes of fiber and some micronutrients [10, 14–19].

Cross-sectional and prospective studies have also found that frequent FAFH consumption is associated with higher body mass index (BMI) and percent body fat as well as increased risk for overweight/obesity, cardiometabolic risk factors, and type 2 diabetes [7, 10, 15, 16, 19–40]. The Coronary Artery Risk Development in Young Adults (CARDIA) study found that change in fast-food frequency over 15 years was directly associated with changes in body weight [41]. Similarly, a national study of Australian young adults reported that, after adjusting for confounding variables including age, leisure time physical activity, TV viewing, and employment status, consuming takeaway food twice a week or more was associated with a 31% higher prevalence of moderate abdominal obesity in men and a 25% higher prevalence in women, compared to those who ate takeaway food less than twice a week [39]. Many of the studies linking FAFH with overweight and obesity were conducted with children, adolescents, or young adults [9, 10, 28, 29, 34, 36–39, 42–44].

The shift that has occurred in consumption frequency of FAFH has had important implications in terms of diet quality, including fruit and vegetable intake, and obesity. This cross-sectional study aimed to examine the relationship between FAFH frequency and fruit and vegetable intake as well as the relationship between FAFH frequency and BMI among a representative sample of adult men and women King County, Washington. As most previous studies have focused on younger age groups, the present study fills an important gap in the literature.

## 2. Methods

The Seattle Obesity Study was a cross-sectional study of 2,001 adult male and female residents of King County, Washington. A stratified random sampling scheme was used to ensure a representative sample by income and race/ethnicity. Detailed study methodology has previously been published [45, 46] and discussed here briefly. To develop the sampling frame, randomly generated telephone numbers were matched with residential addresses, using commercial database. A prenotification letter was mailed out to alert potential respondents that their household had been randomly selected for this study. Telephone calls were made to every household to randomly select an adult member as a survey respondent. Exclusion criteria were age less than 18 years, discordance between addresses and telephone numbers obtained from the commercial database, and those self-reported by the respondent. After screening for eligibility, study procedures were explained to the potential respondent and a verbal consent was obtained over the phone and formally recorded. A 25-minute telephone survey was then administered to all the participants. A total of 2,001 respondents participated in the telephone survey. The SOS sample was demographically comparable to 2007 Behavioral Risk Factor Surveillance System (BRFSS) and King County Census data. All procedures involving human subjects/patients were approved by The University of Washington Institutional Review Board.

### 2.1. Frequency of Food Consumption Away from Home (FAFH)

Self-reported data on frequency of FAFH was available from 1,976 of 2,001 phone survey respondents. Respondents were asked to report number of meals eaten out each week, including breakfast and lunch. Frequency of FAFH was categorized as 0-1, 2–4, or 5 or more times per week based on the distribution of the data obtained.

### 2.2. Sociodemographic and Lifestyle Measures

The telephone survey asked a series of questions to collect self-reported data on socioeconomic status (SES) and demographic and lifestyle variables. Annual household income and education were used as the indicators of SES. Income data collected using 9 categories was recombined into three categories for analytical purpose: <$50,000, $50,000–$100,000, and >$100,000, herein $50K, $50–$100K, and >$100K. The 6-category education variable was recombined into three groups: “high school or less,” “some college,” and “college graduates or higher.” Demographic variables of interest were age, gender, race/ethnicity, and household size. Smoking and physical activity were used as lifestyle indicators. Physical activity was captured using the standard question from BRFSS (Behavioral Risk factor Surveillance System)—“During the past month, other than your regular job, did you participate in any physical activity or exercise in your leisure time apart from your work?” For analytical purpose, it was treated as a dichotomous variable: yes/no. The smoking variable was dichotomized into never versus current/former. One thousand six hundred ninety-seven respondents had complete data for the demographic and lifestyle variables.

### 2.3. Health Outcome Measure: BMI

Self-reported data on weight and height was available from 1,877 survey respondents and was used to compute BMI. BMI was calculated as weight (in kilograms) divided by square of height (in meters).

### 2.4. Fruit and Vegetable Intake

Standard dietary questions from BRFSS were used to capture the intake of fruits and vegetables, and other foods for the present study. Each respondent was asked to report the frequency of consumption of fruit juices, fruits, green salad, carrots, and other vegetables by per day/week/month or year basis. The reported frequency for each of these items was added together to compute the total fruit and vegetable intake per person per day. Fruit and vegetable intake data were available from 1,937 respondents.

### 2.5. Statistical Analyses

All statistical analyses were conducted using SPSS 21.0 for Windows (version 2012, IBM-SPSS, Inc.). First, univariate descriptive statistics were conducted for each variable (means, standard deviations, and frequencies). Bivariate associations were then examined between frequency of FAFH and each of the sociodemographic variables, BMI, and fruit and vegetable consumption, separately for males and females as well as for both genders combined. Chi-square tests of independence or analysis of variance tests, followed by post hoc comparisons, were run to test the association of these demographic variables with FAFH. Finally, General Linear Models (GLM) were used to assess the relationship between BMI and FAFH, and fruit and vegetable consumption and FAFH. The analyses were conducted separately for each gender as well as for both genders combined. The dependent variables were, respectively, BMI and fruit and vegetable consumption. The independent variables tested in the model were FAFH, gender, age, education, income, marital status, physical activity, and smoking. For the model for both genders combined, interaction terms between gender and other relevant independent variables were tested.

## 3. Results

Sample characteristics of the 1570 adults with complete data for all of the above described variables are presented in Table 1. A higher percentage of females participated than males (60% versus 40%, resp.). Mean age of the sample was 53.7. The sample was more likely to be White (84%), educated (57% college graduate), and with annual household income of >$50K (60%). About half the sample was married/member of an unmarried couple (57%), and half of the sample never smoked (53%). Most of the sample reported being physically active outside work (79%). Half of the sample consumed FAFH 0-1 time per week, with 34% of the sample consuming 2–4 times per week, and 16% eating out 5 or more meals per week. Average FV consumption was 4.19 servings per day, and the average BMI was 26.6.

Sociodemographic profile of the sample was also observed by gender (Table 1). Females and males were comparable by income, education, race, marital status, and physical activity. However, differences were observed by other variables of interest. Average BMI was much higher among males (27.3 kg/m^2^) as compared to females (26.1 kg/m^2^). Overall, FAFH frequency was higher among males than females (43% versus 54% eating out 0-1 meal per week, resp.). A significantly higher proportion of males ate out 5 or more meals per week (22% versus 12% among females) (*p* value < 0.001). On average, females reported eating approximately half a serving more of fruits and vegetables per day than males (4.45 versus 3.81 servings per day, resp.; *p* < 0.001).

Bivariate associations between FAFH frequency and key sociodemographic variables are presented in Table 2. Significant bivariate trends were observed in frequency of FAFH with selected sociodemographic variables, health indicators, and FV consumption. Higher frequency of FAFH was associated with being younger in age and higher BMI both among females and males (*p* < 0.05). For example, females with FAFH frequency 5+ times per week were on average 50 y old as compared to 57 y old for those never or rarely eating out (defined as 0-1 time per week). Higher frequency of FAFH was associated with relatively higher BMI both among females and males. For example, mean BMI was 27.4 kg/m^2^ among females with frequency of FAFH 5+ times/week as compared to 25.8 kg/m^2^ among those who never or rarely ate out (0-1 time per week) (*p* < 0.05). For females, frequency of FAFH was higher among college graduates (15% with FAFH frequency 5+ times per week) as compared to those with high school or some college (only 7%). Similar trends were seen among males (25% versus 15%, resp.), although the relationship was not significant. Respondents with higher annual household income had higher frequency of FAFH. For example, among females, 12% of those with household income of $50–$100K and 20% of those with income >$100K ate out five or more times per week as compared to only 7% of those with income <$50K. The corresponding numbers among males were 23%, 28%, and 17%, respectively.

There were mixed results for the associations between FAFH and fruit and vegetable intake. For example, for males, while there were significant differences between fruit and vegetable intake for those who ate out 0-1 or 2–4 times per week versus 5 or more times per week, the overall association was not significant (3.98, 3.78, and 3.53, *p* value = 0.093). A similar pattern was observed for females.

No significant differences were observed in frequency of FAFH by race, marital status, or smoking status.

**Figure 1:**
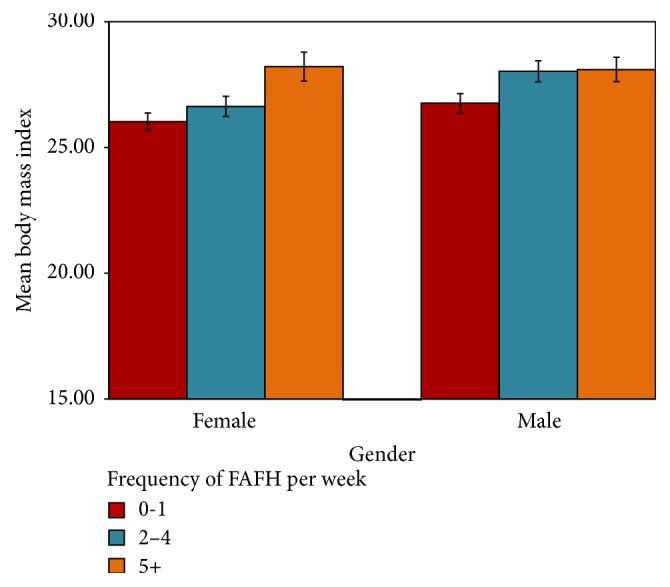
Mean BMI by gender and frequency of foods away from home (FAFH). Mean BMI by gender and frequency of foods away from home (FAFH) after controlling for age, race, gender, education, income, marital status, smoking status, and physical activity.

Table 3 shows associations between BMI and frequency of FAFH, separately among females and males as well as for both genders combined, after adjusting for covariates. There were no significant interactions between gender and other relevant independent variables. Among females, frequency of FAFH 5 or more times per week was associated with significantly higher BMI as compared to those who eat out only once per week or never (*β* = 2.19; SE = 0.60; *p* value < 0.001). However, no significant difference in BMI was observed among females with frequency of FAFH 2–4 times per week as compared to the reference category. Among males, eating out 2–4 times per week and 5 times per week or more were associated with significantly higher BMI (*β* = 1.27; SE = 0.44; *p* value = 0.004 and *β* = 1.34; SE = 0.52; *p* value = 0.010, resp.), as compared to those who only eat out only once a week or never. These associations were observed even after adjusting for covariates: income, education, race/ethnicity, marital status, and lifestyle variables (Figure 1).

Significant associations were also observed between key sociodemographic and lifestyle variables and BMI, both among females and males. For example, higher income was associated with significantly lower BMI among females (as compared to the reference category of <$50K, *β* = 1.87, *p* value 0.001 among those with income $50–$100K; and *β* = 1.35, *p* value 0.008 among those with income >$100K). For males, a significant relation was observed with education. As compared to college graduates, having lower education was associated with higher BMI (*β* = 1.23, *p* value: 0.028). Physical activity had a significant negative association with BMI for both females and males (among females, *β* = 2.61, *p* value ≤ 0.001 for those who were not physically active outside work; among males, *β* = 1.33, *p* value = 0.006). No other effects were significant in the models. For the models with gender combined, eating out 2–4 times per week and 5 or more times per week were associated with significantly higher BMI (*β* = 0.97, *p* value = 0.001 and *β* = 1.88, *p* value < 0.001, resp.), as compared to those who eat out only once a week or never.

Table 4 displays the multivariate associations between FV consumption and frequency of FAFH, separately for females and males. Among females, those who ate FAFH 2–4 times per week consumed significantly fewer servings of fruit and vegetables per day than those who ate out 0-1 time per week (*β* = −0.36; SE = 0.15; *p* value = 0.018), adjusted for income, education, race/ethnicity, marital status, and lifestyle variables. However, no significant difference in FV consumption was observed among females who ate FAFH 5 or more times per week compared to those who ate out 0-1 time per week. Among males, no significant associations were observed between FV consumption and FAFH frequency. There were no significant interactions between gender and other relevant independent variables. For the models with gender combined, eating FAFH 2–4 times per week and 5 or more times per week was associated with significantly less FV consumption (*β* = −0.32, *p* value = 0.007 and *β* = −0.46, *p* value = 0.003, resp.), compared to eating FAFH 0-1 time per week.

## 4. Discussion

### 4.1. FAFH and BMI

In the current study, higher frequency of FAFH was associated with higher BMI among both females and males. A 2012 review reported that 7 out of 8 cohort studies found a positive association between the frequency of eating away from home and body weight [23]. Among cross-sectional studies, the same review reported that, of 27 cross-sectional studies, 9 showed no association or a negative association among women and 11 showed no association or a negative association among men [23]. In those studies that found no relationship between FAFH frequency and body weight, the authors of the review suggested that lack of a common definition of the out-of-home eating concept, food intake assessment method, small sample size, and, in a Spanish study, frequency of consumption of food from healthy restaurants versus fast food establishments may have contributed to the lack of association [23].

### 4.2. Explaining the Relationship between FAFH and BMI

In general, fast foods and FAFH tend to be energy dense. A number of studies have found FAFH to be energy dense, and with higher amounts of total fat and sugar than home-prepared meals [12, 15, 47, 48]. Portion sizes at fast food chains and restaurants might be another factor that contributes to unintended consumption of excess energy. Fast food and restaurants portion sizes have increased over the past 30 years. For example, Young and Nestle found that current sizes for French fries, hamburgers, and sodas are 2 to 5 times larger than the original sizes offered [49]. A number of experiments have determined that people eat more when they are served larger portions [50–56]. Energy content and portion size in foods away from home likely contribute to excess energy intake in those who often eat out. This excess energy intake may lead to weight gain, since individuals often do not compensate for consuming larger portions by decreasing caloric intake or increasing physical activity [50, 54, 55]. Overweight individuals may be more affected by large portion sizes than normal weight consumers [57].

Influences on food choice, including the choices to eat out, eat less, or eat more healthful foods, are complex and include food upbringing, family role, health, ethnic identity and traditions, food environment, and resources, including knowledge and skills; time, space, and finances; social networks and supports; and cultural and social skills [58]. Other models of food choice include explanations of value negotiations, weighing sensory perceptions, monetary considerations, health and nutrition beliefs and concerns, convenience, and social relationships [59]. Recent evidence indicates that a greater amount of time spent on home food preparation is associated with more frequent intake of fruits and vegetables [60, 61].

### 4.3. FAFH Frequency and FV Consumption

Although there were mixed results for the associations between FAFH frequency and FV consumption in this study, the findings suggest a negative correlation. Most studies have found eating out to be associated with poor diet quality, including lower consumption of fruits and vegetables [10, 14–19]. A few previous studies found that eating out was associated with higher vegetable consumption [8, 10, 62]. In one of these, the authors suggested the categorization of French fries as a vegetable may have contributed to this finding [8]. Two studies found that eating at full service restaurants was associated with higher vegetable consumption, but eating at burger-and-fries or non-fast food restaurants was not associated or negatively associated with vegetable consumption [10, 62]. A few studies have found frequency of fruit consumption to be higher among those who ate out more frequently, at least in some subpopulations [62–64]. Befort and colleagues found that, for Black adolescents, fruit intake was positively associated with sit-down, menu-based restaurant use, but this was not the case for White adolescents or for fast food restaurant use [62]. A study of Korean housewives found that, in the middle income class, frequency of fruit consumption was significantly higher with more frequent eating out, but there were no associations in the lower or upper classes [63]. Another Korean study reported that people who ate out more frequently consumed more fruit; the authors noted that this could be explained by Korean restaurants serving fresh fruit as dessert [64].

In the current analysis, eating away from home included eating at full service and fast food restaurants, but, as mentioned, the relationship between frequency of eating away from home and poor diet quality may vary according to restaurant type [10, 62]. That is, food eaten at full service restaurants may differ in key nutrients from food obtained from fast food restaurants. For example, in a study of young adults, Larson and colleagues found more frequent use of full service restaurants was associated with higher vegetable and fiber consumption [10].

### 4.4. Explaining the Relationship between FAFH Frequency and FV Consumption

One of the reasons for the negative relation between FAFH frequency and FV consumption may be the lack of demand for, and thus lack of options offered of, healthy food at fast food restaurants. An Australian study found that, out of nearly 1500 meal purchases at McDonald's stores in a variety of socioeconomic areas, only 1.5% were healthy, which was defined by a symbol denoting approval from the National Heart Foundation of Australia [65]. Another study of fast food patrons found only 58% rated nutrition as important when buying fast food [66]. Likewise, interviews with senior menu development and marketing executives at leading casual dining and fast-food restaurant chains revealed they believe demand for healthier foods is not widespread [67]. Additional obstacles to including healthier menu items mentioned by the executives were the short shelf life of produce, increased preparation time, low sales, and high labor costs [67]. The authors noted that profit margins are the primary determinants of restaurants' choice to continue to serve healthier food options and that without an increase in consumer demand it is unlikely the restaurant industry will increase their offering of healthy food choices [67].

### 4.5. Results by Gender

Overall, in this study's sample, frequency of FAFH was higher among males than females. This finding is consistent with previous research [41, 68, 69]. On average, females in the current study reported eating approximately half a serving more of fruits and vegetables per day than males. This finding is also consistent with previous research [70–72].

### 4.6. Results by Demographic and Lifestyle Factors

In the current study, younger individuals ate out more often than older individuals, a finding supported by previous research [24, 73, 74]. In addition, individuals with higher incomes ate out more frequently. The inclusion of both cheaper and more expensive restaurants in the definition of FAFH in this study may have contributed to the finding that those with higher incomes ate out more frequently. Although eating out is generally associated with eating more unhealthful food and more calories in general, some restaurants offer healthier options and not all consumers eat more at a meal away from home compared to an at-home meal.

### 4.7. Recommendations

Among the recommendations in a 2006 report requested by the FDA were that food establishments should increase the availability of low-calorie menu items, provide consumers with caloric and nutrient information in a standard, easily accessible format, and develop and promote food and beverage portion size options that help consumers in their efforts to balance their energy intake and output [75].

Environmental changes may be an option. A review of worksite health promotion programs found that fruit, vegetable, and fat intake can be positively influenced by environmental strategies including labeling at the point of purchase, promotional materials, expanded availability of healthy foods, and targeted food placement [76]. In school settings, successful environmental strategies for increasing healthy food purchasing include food pricing, availability, and promotion [77]. Restaurant managers interested in promoting healthy food choices, while continuing to make a profit, could implement some of these strategies.

Other strategies concentrate more on consumers making healthy decisions when they choose to eat out. USDA recommendations include selecting water over sugar-sweetened beverages and opting for small or medium portions and dishes that have been steamed, grilled, or broiled versus fried or sautéed [78]. At fast food restaurants, recommendations include using available nutrition information to inform decisions and passing on “super-sizing” [79].

As mentioned, one reason people buy fast food is the notion that it is cheaper than home-prepared food. Articles in the popular press have attempted to refute this idea [80]. If this were true, one reason for eating away from home could be eliminated. Unfortunately, previous analyses of Seattle Obesity Study data suggest that, based on the monetary value of individual diets assessed by food frequency questionnaire, higher-cost diets were significantly higher in nutrient density [46, 81, 82]. Other studies support the finding that higher cost diets are more nutritionally dense [83–86].

### 4.8. Study Limitations

This study has important limitations that must be noted. First, the self-reported nature of the data for the key outcomes of interest is prone to error and bias. Next, due to the cross-sectional nature of the study, causality cannot be determined. It is possible that eating foods away from home more frequently results in higher overall calorie consumption and therefore higher BMI as well as lower fruit and vegetable intake; however, it is also possible that individuals of higher body weight and those who consumer fewer fruits and vegetables are more likely to consume away from home meals. Finally, while this was a representative sample in Seattle, Washington, these findings may not be generalizable to all U.S. cities and/or geographic regions outside the U.S.

## 5. Conclusion

In this study of 1570 adults, a positive relationship was found between FAFH and BMI in both men and women. Those who ate out more frequently tended to consume fewer fruits and vegetables, although the results were not consistent among all analyses. Overall, frequency of FAFH was higher among males than females. On average, females reported eating approximately half a serving more of fruits and vegetables per day than males. Eating out less frequently and choosing healthy, low calorie meals when away from home may help reduce overconsumption of energy-dense, nutrient-poor foods. Recommendations include both food establishments and consumers taking responsibility for decreasing these trends; food establishments could provide and promote healthier foods, while consumers could choose more nutrient-dense options.

## Figures and Tables

**Table 1:** Sample characteristics of female and male adults in the sample.

Variable			Gender			
	Total		Female		Male	

*N*	1570		936 (60%)		634 (40%)	

	Mean ± SD	*n*	Mean ± SD	*n*	Mean ± SD	*n*

Age	53.72 ± 14.64	1570	54.00 ± 14.83	936	53.31 ± 14.34	634
BMI	26.56 ± 5.45	1570	26.09 ± 5.81	936	27.25 ± 4.81	634

	Total		Female		Male	
	*N* (%)		*N* (%)		*N* (%)	

Race						
White	1313 (84)		782 (84)		531 (84)	
Not White	257 (16)		154 (17)		103 (16)	
Education						
High school	263 (17)		154 (17)		109 (17)	
Some college	410 (26)		243 (26)		167 (26)	
College graduate	897 (57)		539 (58)		358 (57)	
Income						
<$50K	617 (39)		386 (41)		231 (36)	
$50–$100K	540 (34)		318 (34)		222 (35)	
>$100K	413 (26)		232 (25)		181 (29)	
Marital status						
Married or member of unmarried couple	891 (57)		521 (56)		370 (58)	
Divorced/separated/widowed/never married	679 (43)		415 (44)		264 (42)	
Smoking status						
Never smoker	838 (53)		527 (56)		311 (49)	
Former/current smoker	732 (47)		409 (44)		323 (51)	
Physical activity						
Yes	1238 (79)		732 (78)		506 (80)	
No	332 (21)		204 (22)		128 (20)	
Frequency of FAFH per week (FAFH)						
0-1	778 (50)		505 (54)		273 (43)	
2–4	539 (34)		319 (34)		220 (35)	
5+	253 (16)		112 (12)		141 (22)	

	Mean ± SD	*n*	Mean ± SD	*n*	Mean ± SD	*n*

Fruit and vegetable servings per day	4.19 ± 2.13	1570	4.45 ± 2.18	936	3.81 ± 2.01	634

FAFH, foods away from home.

**Table 2:** Bivariate associations between FAFH, key sociodemographic variables, and fruit and vegetable consumption.

	Total				Female				Male			
	0-1	2–4	5+	*p* value	0-1	2–4	5+	*p* value	0-1	2–4	5+	*p* value
Age mean (SE)	56.57 (0.52)	51.37 (0.62)	49.95 (0.90)	<0.001	56.77 (0.65)	51.11 (0.81)	49.72 (1.37)	<0.001	56.20 (0.86)	51.76 (0.95)	50.12 (1.19)	<0.001
BMI mean (SE)	26.07 (0.20)	26.77 (0.23)	27.62 (0.34)	<0.001	25.78 (0.26)	26.11 (0.32)	27.44 (0.55)	0.024	26.59 (0.29)	27.73 (0.32)	27.76 (0.40)	0.012

Race												
White count (%)	632 (48)	464 (35)	217 (17)	0.039	411 (53)	277 (35)	94 (12)	0.120	221 (42)	187 (35)	123 (23)	0.214
Not White	146 (57)	75 (29)	36 (14)		94 (61)	42 (27)	18 (12)		52 (51)	33 (32)	18 (18)	
Education												
High school	159 (61)	77 (29)	27 (10)	<0.001	102 (66)	41 (27)	11 (7)	<0.001	57 (52)	36 (33)	16 (15)	0.166
Some college	215 (52)	139 (34)	56 (14)		145 (60)	79 (33)	19 (8)		70 (42)	60 (36)	37 (22)	
College graduate	404 (45)	323 (36)	170 (19)		258 (48)	199 (37)	82 (15)		146 (41)	124 (35)	88 (25)	
Income												
<$50K	371 (60)	181 (29)	65 (11)	<0.001	255 (66)	105 (27)	26 (7)	<0.001	116 (50)	76 (33)	39 (17)	0.003
$50–$100K	250 (46)	199 (37)	91 (17)		151 (48)	128 (40)	39 (12)		99 (45)	71 (32)	52 (23)	
>$100K	157 (38)	159 (39)	97 (24)		99 (43)	86 (37)	47 (20)		58 (32)	73 (40)	50 (28)	
Marital status												
Married/relationship^a^	439 (49)	312 (35)	140 (16)	0.770	270 (52)	184 (35)	67 (13)	0.317	169 (46)	128 (35)	73 (20)	0.140
Single^b^	339 (50)	227 (33)	113 (17)		235 (57)	135 (33)	45 (11)		104 (39)	92 (35)	68 (26)	
Smoking status												
Never smoker	415 (50)	285 (34)	138 (17)	0.908	295 (56)	166 (32)	66 (13)	0.166	120 (39)	119 (38)	72 (23)	0.071
Current/former smoker	363 (49.6)	254 (35)	115 (16)		210 (51)	153 (37)	46 (11)		153 (47)	101 (31)	69 (21)	
Physical activity												
Yes	592 (50)	448 (36)	198 (16)	0.009	384 (53)	260 (36)	88 (12)	0.179	208 (41)	188 (37)	110 (22)	0.033
No	186 (56)	91 (27)	55 (17)		121 (59)	59 (29)	24 (12)		65 (51)	32 (25)	31 (24)	

Fruit & veg. servings per day	4.38 (0.08)	4.08 (0.09)	3.86 (0.13)	0.001	4.59 (0.10)	4.28 (0.12)	4.27 (0.21)	0.091	3.98 (0.12)	3.78 (0.14)	3.53 (0.17)	0.093

FAFH, foods away from home.

^a^Married or member of unmarried couple.

^b^Divorced/separated/widowed/never married.

**Table 3:** Multivariate analysis of BMI and frequency of FAFH by gender, adjusted for covariates.

Dependent variable: body mass index (BMI)	Total			Female			Male		
Parameter	*β*	SE	*p* value	*β*	SE	*p* value	*β*	SE	*p* value
Intercept	24.28	0.89	<0.001	23.27	1.18	<0.001	25.44	1.34	<0.001
Age	0.01	0.01	0.314	0.01	0.02	0.406	0.01	0.02	0.550
Race (ref. = White)									
Not White	−0.72	0.37	0.052	−0.68	0.50	0.171	−0.92	0.54	0.089
Education (ref. = college graduate)									
High school	0.68	0.40	0.090	0.13	0.56	0.811	1.24	0.56	0.028
Some college	0.22	0.33	0.514	0.05	0.45	0.918	0.41	0.47	0.384
Income (ref. ≥ $100K)									
<$50K	1.26	0.41	0.002	1.87	0.58	0.001	0.66	0.56	0.242
$50–$100K	0.93	0.36	0.010	1.36	0.51	0.008	0.54	0.50	0.281
Marital status (ref. = married or member of unmarried couple)									
Divorced/separated/widowed/never married	−0.37	0.33	0.271	−0.11	0.45	0.803	−0.72	0.49	0.143
Smoking status (ref. = current/former smoker)									
Never smoked	−0.35	0.27	0.200	−0.58	0.38	0.126	0.13	0.40	0.741
Physical activity (ref. = yes)									
No	2.07	0.34	<0.001	2.61	0.46	<0.001	1.33	0.48	0.006
Frequency of FAFH per week (FAFH) (ref. = 0-1)									
2–4	0.97	0.30	0.001	0.61	0.41	0.140	1.27	0.44	0.004
5+	1.88	0.40	<0.001	2.19	0.60	<0.001	1.34	0.52	0.010

FAFH, foods away from home; ref., reference category.

**Table 4:** Multivariate analysis of FV servings per day and frequency of FAFH by gender, adjusted for covariates.

Dependent variable: fruit and vegetables servings/day	Total			Female			Male		
Parameter	*β*	SE	*p* value	*β*	SE	*p* value	*β*	SE	*p* value
Intercept	3.56	0.34	<0.001	3.663	0.44	<0.001	3.54	0.55	<0.001
Age	0.02	0.00	<0.001	0.018	0.01	0.001	0.01	0.01	0.065
Race (ref. = White)									
Not White	0.32	0.14	0.028	0.40	0.19	0.033	0.19	0.22	0.396
Education (ref. = college graduate)									
High school	−0.74	0.16	<0.001	−0.97	0.21	<0.001	−0.37	0.23	0.105
Some college	−0.61	0.13	<0.001	−0.46	0.17	0.007	−0.81	0.19	<0.001
Income (ref. ≥ $100K)									
<$50K	0.27	0.16	0.095	0.31	0.22	0.157	0.20	0.23	0.384
$50–$100K	0.45	0.14	0.001	0.53	0.19	0.006	0.36	0.20	0.079
Marital status (ref. = married or member of unmarried couple)									
Divorced/separated/widowed/never married	−0.14	0.13	0.298	−0.16	0.17	0.358	−0.22	0.20	0.263
Smoking status (ref. = current/former smoker)									
Never smoked	−0.04	0.11	0.740	−0.08	0.14	0.565	−0.10	0.16	0.540
Physical activity (ref. = yes)									
No	−0.93	0.13	<0.001	−1.04	0.17	<0.001	−0.77	0.20	<0.001
Frequency of FAFH per week (FAFH) (ref. = 0-1)									
2–4	−0.32	0.12	0.007	−0.36	0.15	0.018	−0.16	0.18	0.386
5+	−0.46	0.15	0.003	−0.30	0.23	0.182	−0.34	0.21	0.106

FV, fruit and vegetable; FAFH, foods away from home; ref., reference category.

## Abbreviations

BMI: Body mass index
FAFH: Foods away from home
FV: Fruit and vegetable
NHANES: National Health and Nutrition Examination Survey
CSFII: Continuing Survey of Food Intakes by Individuals
BRFSS: Behavioral Risk factor Surveillance System
CARDIA: Coronary Artery Risk Development in Young Adults
SES: Socioeconomic status.
